# Supplementary material for: The acyl- and alkyl-glycerone phosphate reductase DHRS7 is involved in the production of distinct plasmalogen species from DHRS7B
Source: J Cell Sci. 2026 May 5;139(9):jcs264759. doi: 10.1242/jcs.264759 (PMC13200781; doi:10.1242/jcs.264759)
Supplement: Supplementary information [file joces-139-264759-s1.pdf]

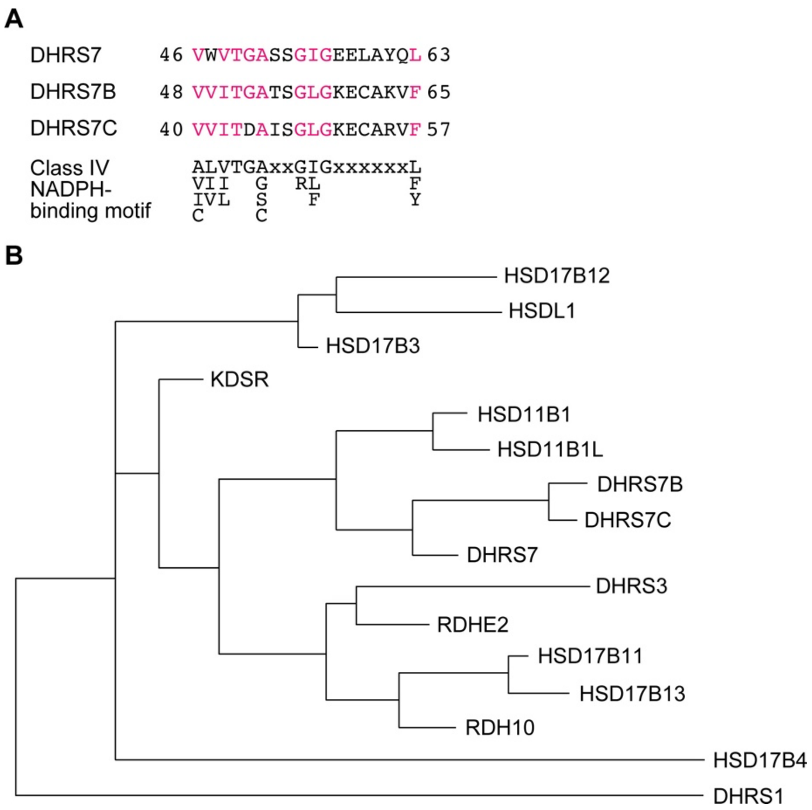

Fig. S1. NADPH-binding motif alignment and phylogenetic tree of DHRS7 subfamily proteins. (A) Alignment of NADPH-binding motifs from DHRS7 subfamily proteins. Amino acid residues shown in magenta indicate those that match the consensus class IV NADPH-binding motif sequence. The numbers on the left and right indicate the residue positions in each protein. (B) Phylogenetic tree of the DHRS7 subfamily and closely related SDR family proteins generated by the maximum likelihood method using the Clustal Omega program (https://www.ebi.ac.uk/Tools/msa/clustalo/).

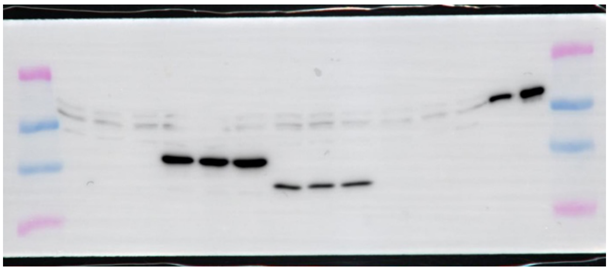

Fig. S2. Full, uncropped immunoblot image corresponding to Fig. 6A. The uncropped immunoblot corresponding to Fig. 6A is shown. The two lanes with colored blue and pink bands represent molecular weight marker lanes. Lanes 1–3 contain lysates from vector-transfected cells, lanes 4–6 from 3×FLAG-DHRS7-expressing cells, lanes 7–9 from 3×FLAG-DHRS7B-expressing cells, and lanes 10–14 contain samples unrelated to the present experiment but are included because the entire membrane is shown without cropping.

Journal of Cell Science • Supplementary information

Table S1. Sequences of primers and oligonucleotides used in this study.

Name	Sequence
mDhrs7_F1	5′-GTGACTGTGAACAGCATCGC-3′
mDhrs7_R1	5′-TGGATATTGGCCGAGCTCAC-3′
mDhrs7b_F1	5′-TGCGTGCAGAGATGGAAGAG-3′
mDhrs7b_R1	5′-CCTGGGCTGTGTTCTTGTCT-3′
mDhrs7c_F1	5′-GAACAGGCCAGATTGTGTTAGTG-3′
mDhrs7c_R1	5′-CCACAACATCGTATTCCTCAACC-3′
mHprt1_F1	5′-GCTGACCTGCTGGATTACATTAAAG-3′
mHprt1_R1	5′-CTTAACCATTTTGGGGCTGTACTGC-3′
hDHRS7_F1	5′-TAATGGAGACCAGTCCCACAAGATG-3′
hDHRS7_R1	5′-GTCTGCATCCACACCACTCTTAAAG-3′
hDHRS7B_F1	5′-TGAAATTGAGGTGACCGTCATCAGC-3′
hDHRS7B_R1	5′-ATAAACAGCCAAGGAAGGCAGTAAG-3′
hDHRS7C_F1	5′-CACATTGACGAAAGCCCTGCTTCCC-3′
hDHRS7C_R1	5′-CGTGGTACGACCGGATGAAAGTCGG-3′
hGAPDH_F1	5′-CCAAGGTCATCCATGACAACTTTGG-3′
hGAPDH_R1	5′-GGTCCACCACCCTGTTGCTGTAGCC-3′
hDHRS7_F2	5′-CACCGATCCATGCACAGAGAACGCT-3′
hDHRS7_R2	5′-AAACAGCGTTCTCTGTGCATGGATC-3′
hDHRS7B_F2	5′-CACCGCTCTGTGGCCGGAATGGTG-3′
hDHRS7B_R2	5′-AAACCACCATTCCGGCCACAGAGC-3′
hDHRS7C_F2	5′-CACCGCGCTGATCAAGGCATCATAT-3′
hDHRS7C_R2	5′-AAACATATGATGCCTTGATCAGCGC-3′
hDHRS7_F3	5′-ACCAACCATCCATAGGAATTCCATC-3′
hDHRS7_R3	5′-CACATCACTTCCAGTTCCAAGGACC-3′
hDHRS7B_F3	5′-CACTGAGGAAGAATGGCTGTCAAGG-3′
hDHRS7B_R3	5′-TTTCTCAACTCACAGTGCTAGCCAG-3′
hDHRS7C_F3	5′-ACCTCGTGGTGCTAAGACTTCTGAG-3′
hDHRS7C_R3	5′-TCCTCTGTCTTCTGGATCCTTGTTG-3′
hDHRS7_F4	5′-TAGGATCCATGAACTGGGAGCTGCTGCTGTGGC-3′
hDHRS7_R4	5′-TATCAGTCATGTTTTGTCTTAAAGATTTTAAA-3′
hDHRS7B_F4	5′-TAGGATCCATGGTCTCTCCGGCTACCAGGAAGAG-3′
hDHRS7B_R4	5′-TACTAGGAGTTCTTGGATTTCCGCTC-3′

Journal of Cell Science • Supplementary information

Table S2. The *m/z* values of precursor and product ions, cone voltages, and collision energies used for the detection of PE[P] and PE species in LC–MS/MS analysis.

PE[P] and PE species	Precursor ion (Q1) [M−H]$^-$	Product ion (Q3)	Cone voltage (V)	Collision energy (eV)
P-C16:0/d_9-C16:0	683.4	264.2	18	34
P-C16:0/C16:0	674.4	255.2	18	34
P-C16:0/C18:0	702.4	283.2	18	34
P-C16:0/C18:1	700.4	281.2	18	34
P-C16:0/C18:2	698.4	279.2	18	34
P-C16:0/C20:4	722.3	303.2	18	28
P-C16:0/C22:4	750.3	331.2	18	28
P-C16:0/C22:6	746.3	327.2	18	28
P-C16:1/C16:0	672.4	255.2	18	34
P-C16:1/C18:0	700.4	283.2	18	34
P-C16:1/C18:1	698.4	281.2	18	34
P-C16:1/C18:2	696.4	279.2	18	34
P-C16:1/C20:4	720.3	303.2	18	28
P-C16:1/C22:4	748.3	331.2	18	28
P-C16:1/C22:6	744.3	327.2	18	28
P-C16:2/C16:0	670.3	255.2	18	34
P-C16:2/C18:0	698.4	283.2	18	34
P-C16:2/C18:1	696.4	281.2	18	34
P-C16:2/C18:2	694.3	279.2	18	34
P-C16:2/C20:4	718.3	303.2	18	28
P-C16:2/C22:4	746.3	331.2	18	28
P-C16:2/C22:6	742.3	327.2	18	28
P-C18:0/C16:0	702.4	255.2	18	34
P-C18:0/C18:0	730.4	283.2	18	34
P-C18:0/C18:1	728.4	281.2	18	34
P-C18:0/C18:2	726.4	279.2	18	34
P-C18:0/C20:4	750.3	303.2	18	28
P-C18:0/C22:4	778.4	331.2	18	28
P-C18:0/C22:6	774.3	327.2	18	28
P-C18:1/C16:0	700.4	255.2	18	34

P-C18:1/C18:0	728.4	283.2	18	34
P-C18:1/C18:1	726.4	281.2	18	34
P-C18:1/C18:2	724.4	279.2	18	34
P-C18:1/C20:4	748.3	303.2	18	28
P-C18:1/C22:4	776.4	331.2	18	28
P-C18:1/C22:6	772.3	327.2	18	28
P-C18:2/C16:0	698.4	255.2	18	34
P-C18:2/C18:0	726.4	283.2	18	34
P-C18:2/C18:1	724.4	281.2	18	34
P-C18:2/C18:2	722.4	279.2	18	34
P-C18:2/C20:4	746.3	303.2	18	28
P-C18:2/C22:4	774.3	331.2	18	28
P-C18:2/C22:6	770.3	327.2	18	28
d_{35}-P-C18:0/C16:0	737.6	255.2	18	34
d_{35}-P-C18:0/C18:0	765.6	283.2	18	34
d_{35}-P-C18:0/C18:1	763.6	281.2	18	34
d_{35}-P-C18:0/C20:4	785.5	303.2	18	28
d_{35}-P-C18:0/C22:4	813.5	331.2	18	28
d_{35}-P-C18:0/C22:6	809.5	327.2	18	28
C16:0–C16:0	690.3	255.2	20	38
C16:0–C16:1	688.3	255.2	20	38
C16:0–C18:0	718.4	283.2	20	38
C16:0–C18:1	716.3	281.2	20	38
C16:0–C18:2	714.3	279.2	20	38
C16:0–C20:4	738.3	303.6	20	32
C16:0–C22:4	766.3	331.6	20	32
C16:0–C22:6	762.3	327.6	20	32
C16:1–C18:0	716.3	283.2	20	38
C16:1–C18:1	714.3	281.2	20	38
C16:1–C18:2	712.3	279.2	20	38
C18:0–C18:0	746.4	283.2	20	38
C18:0–C18:1	744.4	281.2	20	38
C18:0–C18:2	742.4	279.2	20	38
C18:0–C20:4	766.3	303.6	20	32

Journal of Cell Science • Supplementary information

C18:0–C22:4	794.4	331.6	20	32
C18:0–C22:6	790.3	327.6	20	32
C18:1–C16:0	716.3	255.2	20	38
C18:1–C18:1	742.4	281.2	20	38
C18:1–C18:2	740.3	279.2	20	38
C18:1–C20:4	764.3	303.6	20	32
C18:1–C22:4	792.3	331.6	20	32
C18:1–C22:6	788.3	327.6	20	32
d_{35}-C18:0–C16:0	753.6	255.2	20	38
d_{35}-C18:0–C16:1	751.6	253.2	20	38
d_{35}-C18:0–C18:0	781.6	283.2	20	38
d_{35}-C18:0–C18:1	779.6	281.2	20	38
d_{35}-C18:0–C20:4	801.5	303.2	20	32
d_{35}-C18:0–C22:4	829.6	331.2	20	32
d_{35}-C18:0–C22:6	825.5	327.2	20	32

Journal of Cell Science • Supplementary information

Table S3. The *m/z* values of precursor and product ions, cone voltages, and collision energies used for the detection of acyl/alkyl-GnP and 1-acyl/alkyl-G3P species in LC–MS/MS analysis.

Species	Precursor ion (Q1) $[M-H]^-$	Product ion (Q3)	Cone voltage (V)	Collision energy (eV)
hexadecyl-GnP	393.2	79.0	30	25
1-hexadecyl-G3P	395.2	79.0	30	25
hexadecanoyl-GnP	407.4	79.0	20	25
1-hexadecanoyl-G3P	409.4	79.0	40	35

Journal of Cell Science • Supplementary information
